# Continuous Fabrication of Bioinspired All-Biomass Aerogel Fibers for Thermal Insulation

**DOI:** 10.1007/s40820-026-02332-3

**Published:** 2026-08-10

**Authors:** Meiling Zhou, Qiaoling Xue, Lulu Fu, Beini Zeng, Shengnan Ouyang, Shouwei Zhang, Jinming Zhang, Qingtao Liu, Xungai Wang, Jinfeng Wang

**Affiliations:** 1 National Local Joint Laboratory for Advanced Textile Processing and Clean Production, Wuhan Textile University, Wuhan, 430200 People’s Republic of China; 2 CAS Key Laboratory of Engineering Plastics, Institute of Chemistry, Chinese Academy of Sciences (CAS), Beijing, 100190 People’s Republic of China; 3 State Key Laborratory of Bio-based Fiber Materials, College of Textile Science and Engineering, Zhejiang Sci-Tech University, Hangzhou, 310023 People’s Republic of China

**Keywords:** Biomimetic core–shell structure, Aerogel fibers, Thermal insulation, Biomass, Polar bear hair

## Abstract

Aerogel fibers have been considered as a promising solution for thermal protection textiles due to their high porosity and low thermal conductivity. However, the scalable production of sustainable and mechanical strong aerogel fibers remains a critical challenge. Here, inspired by the porous core–shell structure of polar bear hair, we report a continuous strategy to fabricate all-biomass aerogel fibers featuring an encapsulated core–shell architecture using silk fibroin as the core and cellulose as the shell. This tunable structure, with radially aligned sheet-like pores and adjustable shell thickness, is achieved through hydrogen bond-driven cellulose contraction and alcohol-induced curing of sheet-like silk fibroin. Such a porous architecture effectively suppresses convective heat transfer and promotes a multi-reflective effect for infrared radiation. The resulting fibers exhibit good mechanical robustness with a tensile load of a single aerogel fiber is up to 200 g without reinforcement. Meanwhile, the aerogel fiber maintains high porosity (79.78%), low thermal conductivity (52.4 ± 4.2 mW m^−1^ K^−1^), and low density (0.193 ± 0.01 g cm^−3^). Furthermore, an aerogel glove fabricated from these fibers demonstrates good thermal insulation. These results provide new insights for the design of biomimetic aerogel fibers with core–shell architecture, offering a scalable and eco-friendly pathway toward advanced wearable thermal management.

**Supplementary Information:**

The online version contains supplementary material available at 10.1007/s40820-026-02332-3.

## Highlights

- A scalable continuous method was established for fabricating all-biomass aerogel fibers.
- Hydrogen-bond-driven cellulose shrinkage and alcohol-cured silk fibroin collaboratively enabled the formation of a core-shell aerogel fiber with aligned lamellar pores in the core.
- The hierarchical aerogel fibers exhibit low thermal conductivity (52.4 ± 4.2 mW m^−1^ K^−1^), low density (0.193 ± 0.01 g cm^−3^), excellent mechanical flexibility, wash stability, and effective thermal protection.

**Supplementary Information:**

The online version contains supplementary material available at 10.1007/s40820-026-02332-3.

## Graphical abstract

**Figure:**
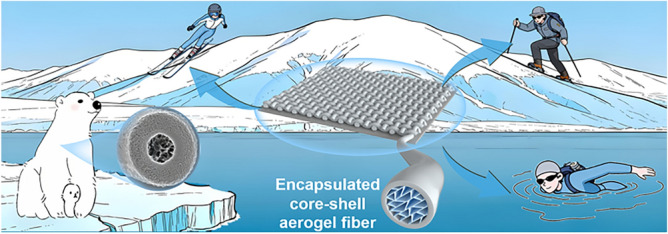

## Introduction

In recent years, flexible thermal-management materials have been increasingly explored for wearable electronics, smart textiles, and personal thermal protection [1–5]. Compared with conventional bulk insulators, fiber-based materials are more compatible with textile systems while offering lightweight characteristics and mechanical flexibility, making them particularly attractive for wearable thermal-management applications [6–8]. Current thermal insulation materials mainly include polymer foams [9], inorganic aerogels [10], and radiative-regulating textiles [11]. Among them, aerogels are particularly effective in suppressing heat transfer because of their highly porous [12–14] and interconnected structures [15, 16]. Nevertheless, conventional inorganic aerogels are generally brittle and difficult to process, whereas many polymer-based insulating materials still face limitations in thermal stability and environmental sustainability. Developing fiber materials that simultaneously combine low thermal conductivity, mechanical flexibility, and sustainability therefore remains challenging [17–19]. Recent advances in asymmetric thermal-management structures, multifunctional biomass-based aerogels, and fire-safe insulating materials have further expanded the design strategies for wearable thermal-management systems [20–22].

The development of biomass-based aerogels has opened new opportunities for sustainable thermal insulation materials. Natural materials such as cellulose [23, 24], chitosan [25], silk fibroin [26], and lignin [27] have been widely used to fabricate lightweight aerogels because of their abundance, biocompatibility, and ability to form hierarchical structures. The abundant micro/nanopores within these aerogels can effectively suppress air convection and thermal diffusion, enabling potential applications in thermal insulation, flexible sensing, electromagnetic shielding, and energy storage. However, most biomass-based aerogels are still fabricated in bulk forms, which often suffer from limited flexibility, low mechanical robustness, and poor compatibility with textile processing, thereby restricting their applications in wearable thermal management [28, 29].

To improve the applicability of aerogels in wearable systems, porous architectures have recently been introduced into continuous fiber structures. Various methods, including freeze spinning [30], wet spinning [31], sol–gel spinning [32], and coaxial spinning [33], have been employed to fabricate aerogel fibers. These fibers retain low density and high porosity while exhibiting favorable flexibility and thermal insulation performance. In addition, biomimetic fibers with core–shell and hierarchical porous structures have gained increasing interest because such architectures can further reduce heat transfer and improve structural stability [34]. Among various biological prototypes, polar bear hair is widely regarded as an ideal model because of its unique hollow core–shell structure, which simultaneously provides excellent thermal insulation, lightweight characteristics, and mechanical flexibility. Inspired by this structure, a variety of polar bear hair-mimetic aerogel fibers have been developed to combine lightweight design with efficient thermal insulation [30, 35, 36]. However, most biomimetic aerogel fibers still rely on multistep fabrication procedures involving solvent exchange, directional freezing, and subsequent drying treatments, which hinders continuous and large-scale production. Moreover, balancing high porosity with sufficient mechanical strength remains a key issue for aerogel fibers.

In this work, we report a continuous fabrication strategy for all-biomass aerogel fibers with a biomimetic self-encapsulated core–shell structure. By integrating coaxial wet spinning, directional freezing, solvent exchange, and ambient-pressure drying, lightweight silk fibroin@cellulose aerogel fibers (SF@CL-AF) with high porosity and low thermal conductivity were directly constructed. The self-encapsulation behavior originates from the shrinkage of the cellulose shell during drying together with the rapid solidification of the silk fibroin-based core in alcoholic solvents. Benefiting from the hierarchical porous architecture, the resulting fibers exhibit favorable thermal insulation, mechanical flexibility, and textile compatibility. This work provides a scalable route toward sustainable aerogel fibers for wearable thermal management and extreme-environment protection.

## Experimental Section

### Materials

Wood pulp was purchased from Dalian Yangrun Trading Co., Ltd. Silkworm cocoons were obtained from the Jiangnan Sericulture Base. BMImOAc was supplied by the Lanzhou Institute of Chemical Physics. Hydrophobic coating was purchased from Zixilai Technology. Na_2_CO_3_, LiBr, and PEG were sourced from Macklin. DMSO and anhydrous ethanol were acquired from Shanghai Pharmaceutical Chemical Reagent Co., Ltd. The chemicals were used as received without further purification.

### Preparation of Pulp Solution and SF Aqueous Solution

Wood pulp solution of 6 wt% was prepared by dissolving wood pulp in a BMImOAc/DMSO solvent mixture (mass ratio of 4:6) at 65 °C under stirring until a transparent solution was formed. Silk cocoons were degummed and dissolved in 9.3 M LiBr solution, which was dialyzed to obtain silk fibroin (SF) aqueous solution and concentrated using PEG to the desired concentration for further use [37].

### Preparation of Biomimetic Aerogel Fibers

Biomimetic aerogel fibers were prepared utilizing the coaxial wet-spinning technique, in which the core layer was composed of a silk fibroin aqueous solution and the shell layer was made of a wood pulp ionic liquid solution. The two solutions were independently delivered into the core and sheath channels of a coaxial nozzle and subsequently gelled within an aqueous coagulation bath to yield wet gel fibers. The wet gel fibers were then subjected to directional freezing to form ice-crystal structure in the internal core of fibers when the fibers go through the low-temperature freezing module [36]. After the freezing step, the frozen fibers were immersed in ethanol for solvent exchange. This process was aimed at curing the sheet-like microstructure of silk fibroin in fiber core and extracting residual water. Finally, the ethanol-saturated wet fibers were dried at 60 °C under ambient pressure to obtain the core–shell structure biomimetic aerogel fibers.

Fibers prepared under various freezing temperatures at − 40, − 60, − 80, and − 100 °C were named as T-40, T-60, T-80, and T-100, respectively. The fibers with core diameter of ~ 610, 660, 710, and 850 μm were name as C-610, C-660, C-710, and C-850, respectively, which were prepared using coaxial needles with inner/outer gauge combinations of 27/15, 24/14, 23/14, and 20/13, respectively. The fibers with shell thickness of ~ 20, 60, 80, and 120 μm, were named as S-20, S-60, S-80, and S-120, respectively, which were prepared using needle pairs of 23/16, 23/14, 23/13, and 23/10, respectively.

### Characterization

The microstructures of the aerogel fibers were observed using a scanning electron microscope (JCM-7000) and a field-emission scanning electron microscope (JEOL JSM-7800F) at an accelerating voltage of 10 kV. Structural parameters of the fibers, including core diameter, shell thickness, and overall diameter, were determined from SEM images. Each parameter was measured at least 10 times independently to ensure statistical reliability. Mechanical properties were evaluated using a universal testing machine (MTS Exceed 44.304) in tensile mode, with a loading rate of 10 mm min^−1^ and a gauge length of 10 mm. At least five specimens with length of 30 mm were tested under each condition to obtain average values and standard deviations. Thermal imaging was performed using a thermal imager (HIKMICRO H10S) at a working distance of approximately 30 cm. Temperature was monitored in real time via a thermocouple connected to a temperature controller (K-612A/B/C). Thermal conductivity was measured using a custom-built steady-state testing platform, where temperature distributions recorded by thermocouples were used to calculate the thermal conductivity coefficient. The optical properties of textiles in the infrared region were examined using a Fourier-transform infrared spectrometer (Vertex 70 FT-IR and Nicolet is50). Hydrophobicity of textile was assessed using a contact angle goniometer (DSA30S). Fiber density was determined using a mass-based pycnometer method using distilled water as the immersion medium. Fiber volume was calculated from the mass of displaced water in the pycnometer, and fiber density was obtained by dividing the fiber mass by the calculated volume.

## Results and Discussion

### Fabrication of Biomimetic Aerogel Fibers

**Fig. 1:**
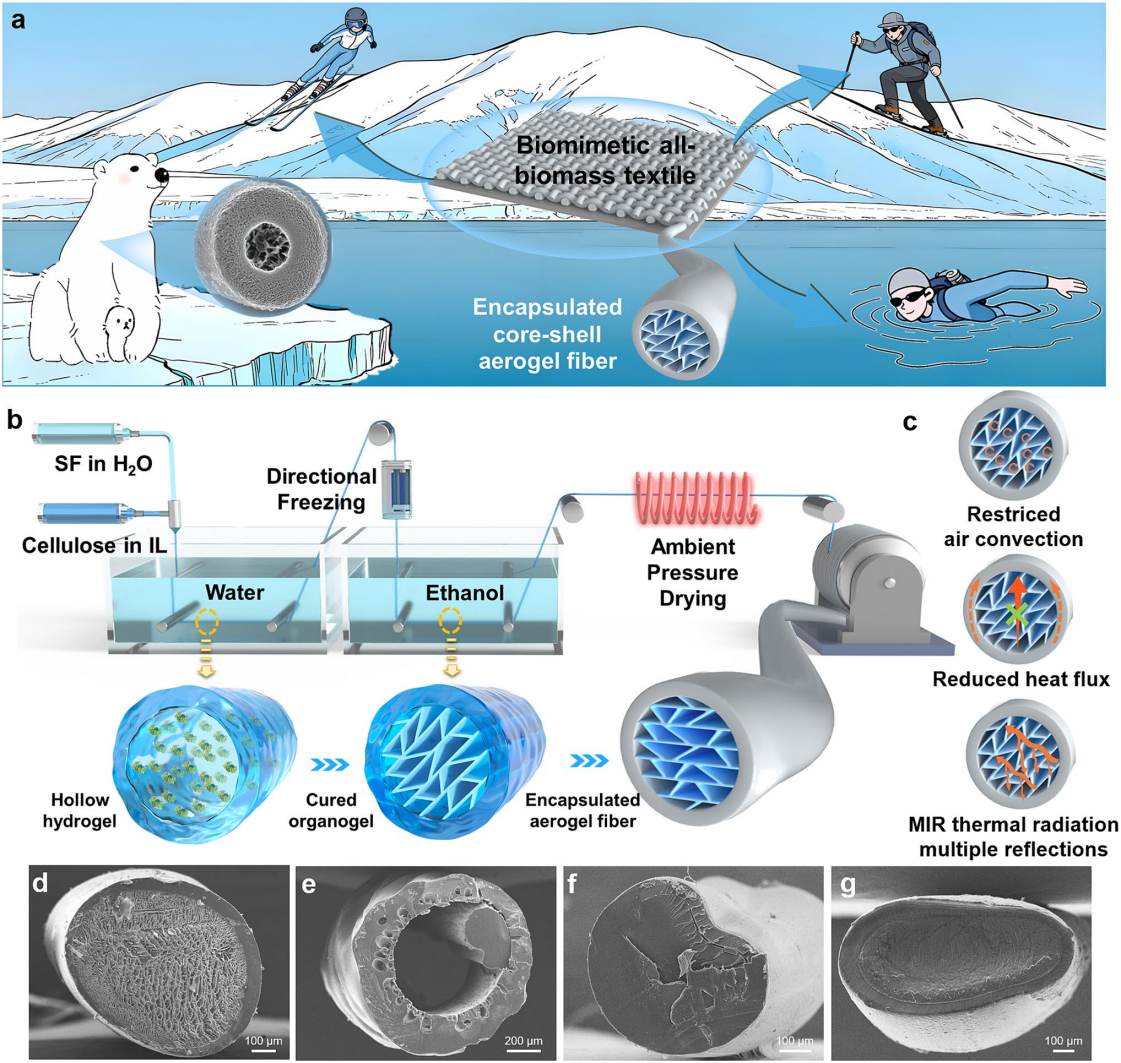
Design and fabrication of biomimetic aerogel fibers. **a** Illustration of thermal insulation performance of polar bear hair and biomimetic aerogel fibers. **b** Fabrication process of aerogel fibers including coaxial wet spinning, directional freezing, solvent exchange, and ambient-pressure drying. **c** Schematic of the thermal insulation mechanism in core–shell aerogel fibers. Ambient pressure dried core–shell fibers under various conditions: **d** self-encapsulated SF-core/cellulose-shell aerogel fiber; **e** cellulose-core/SF-shell fiber; **f** SF-core/cellulose-shell fiber without directional freezing; **g** SF-core/cellulose-shell fiber without ethanol curing

A coaxial wet-spinning method was used to co-extrude an aqueous silk fibroin solution as the core layer and a cellulose ionic liquid solution as the shell layer (Fig. 1b). The cellulose-shell layer underwent rapid gelation in a deionized water coagulation bath. This was followed by directional freezing, which induced ice templating and resulted in the formation of a sheet-like microstructure within the core [35, 36, 38–41]. The frozen fibers then underwent solvent exchange in alcohol-based solvents (e.g., ethanol), which dehydrated the structure and cured the sheet-like aerogel core. Ambient-pressure drying subsequently caused the cellulose shell to contract, spontaneously encapsulating the aerogel core and yielding the core–shell structured aerogel fiber (Fig. 1b–d). In contrast, reversing the core–shell configuration by placing cellulose in the core and silk fibroin in the shell resulted in the cellulose core undergoes substantial shrinkage and collapses into a dense fiber-like structure, whereas the silk fibroin shell largely maintains its external morphology. This structural mismatch led to phase separation and ultimately failed to form the desired aerogel architecture (Fig. 1e). Control experiments further clarified the essential roles of directional freezing and ethanol-induced curing in forming the sheet-like porous structure (Fig. 1f, g). Without directional freezing, the SF-core solidified directly during alcohol treatment, producing a coagulated dense core (Figs. 1f and S2). Omitting ethanol curing caused the sheet-structured SF-core to collapse and densify (Figs. 1g and S2). By strategically integrating the core–shell design with precisely controlled gelation and drying pathways, this process enables the continuous fabrication of aerogel fibers under ambient pressure. Collectively, these findings demonstrate that both the architectural configuration and the coupled freezing-curing protocol are indispensable for producing continuous, porous, and mechanically robust aerogel fibers.

**Fig. 2:**
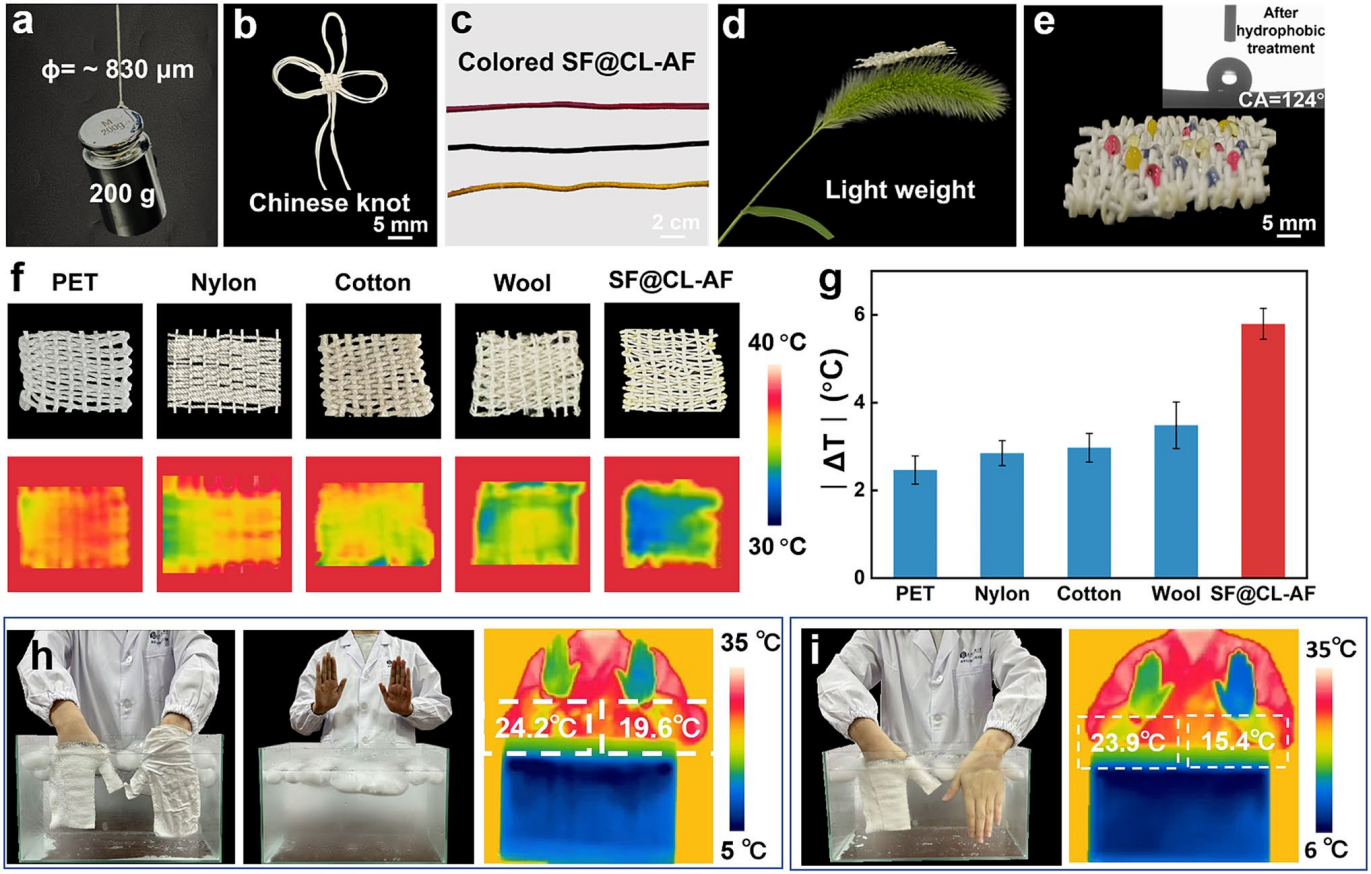
Structural processability and performance of SF@CL aerogel fibers (SF@CL-AF) and textiles. **a** Photograph of a single aerogel fiber under a tensile load of 200 g. Φ denotes the fiber diameter. **b** Chinese knot made from flexible aerogel fibers. **c** Optical image of aerogel fibers in various colors. **d** Aerogel fabric supported on ultralight foxtail grass. **e** Colored water droplets on the superhydrophobic fabric surface. **f** Optical and infrared thermal images of fabrics made from commercial fibers and the aerogel textile placed on a hot stage at 40 °C. **g** Surface-hot stage temperature differences (|ΔT|) of the five textile samples. **h** Optical and infrared images of hands with the superhydrophobic SF@CL-AF and cotton gloves after cold-water immersion. **i** Optical and infrared images of a bare hand and an SF@CL-AF-gloved hand after cold-water immersion for 1 min

The fabricated polar bear hair-inspired aerogel fibers exhibit remarkable flexibility, structural stability, and thermal insulation performance. A single aerogel fiber can sustain a tensile load of up to 200 g without external reinforcement (Fig. 2a), confirming its good load-bearing capacity and structural integrity. Such mechanical robustness provides a solid foundation for weaving and integration into wearable systems. Benefiting from their high flexibility, the fibers can be woven into intricate patterns such as Chinese knots (Fig. 2b). Colorful aerogel fibers can be also prepared by adding various dyes in the spinning solution (Fig. 2c). Owing to the low density of SF@CL-AF (0.193 ± 0.01 g cm⁻^3^), the aerogel textiles can rest stably on delicate supports such as foxtail grass (Fig. 2d). The fabric can be also further surface functionalized to achieve superhydrophobicity. Figure 2e shows that colored water droplets retain a nearly spherical shape on the fiber surface after such treatment indicating excellent water repellency. A bioinspired feature reminiscent of polar bear hair, making the fabric ideal for cold and humid environments. The thermal conductivity of SF@CL-AF is only 52.4 ± 4.2 mW m⁻^1^ K⁻^1^, which is lower than PET, nylon, cotton, and wool (Supplementary Table S1). Thermal insulation performance was also verified by infrared thermal images of fabrics (PET, nylon, cotton, and wool) and the SF@CL-AF textile by placing samples on a 40 °C hot stage. Results show that the aerogel fabric maintained a lower surface temperature than textiles from compared fiber types (Fig. 2f). The quantified surface temperature differences (|ΔT|) under heating conditions further confirm the insulation capability of the aerogel textile (Fig. 2g). This thermal retention was further validated under cold conditions in a cold-water immersion test. The aerogel glove could effectively minimize heat loss with the covered hand maintained a higher surface temperature (around 24 °C) compared to hand wearing cotton gloves (19.6 °C) and the bare hand (15.4 °C) in infrared images (Fig. 2h, i).

### Structural Design of Encapsulated Core–Shell Aerogel Fibers

**Fig. 3:**
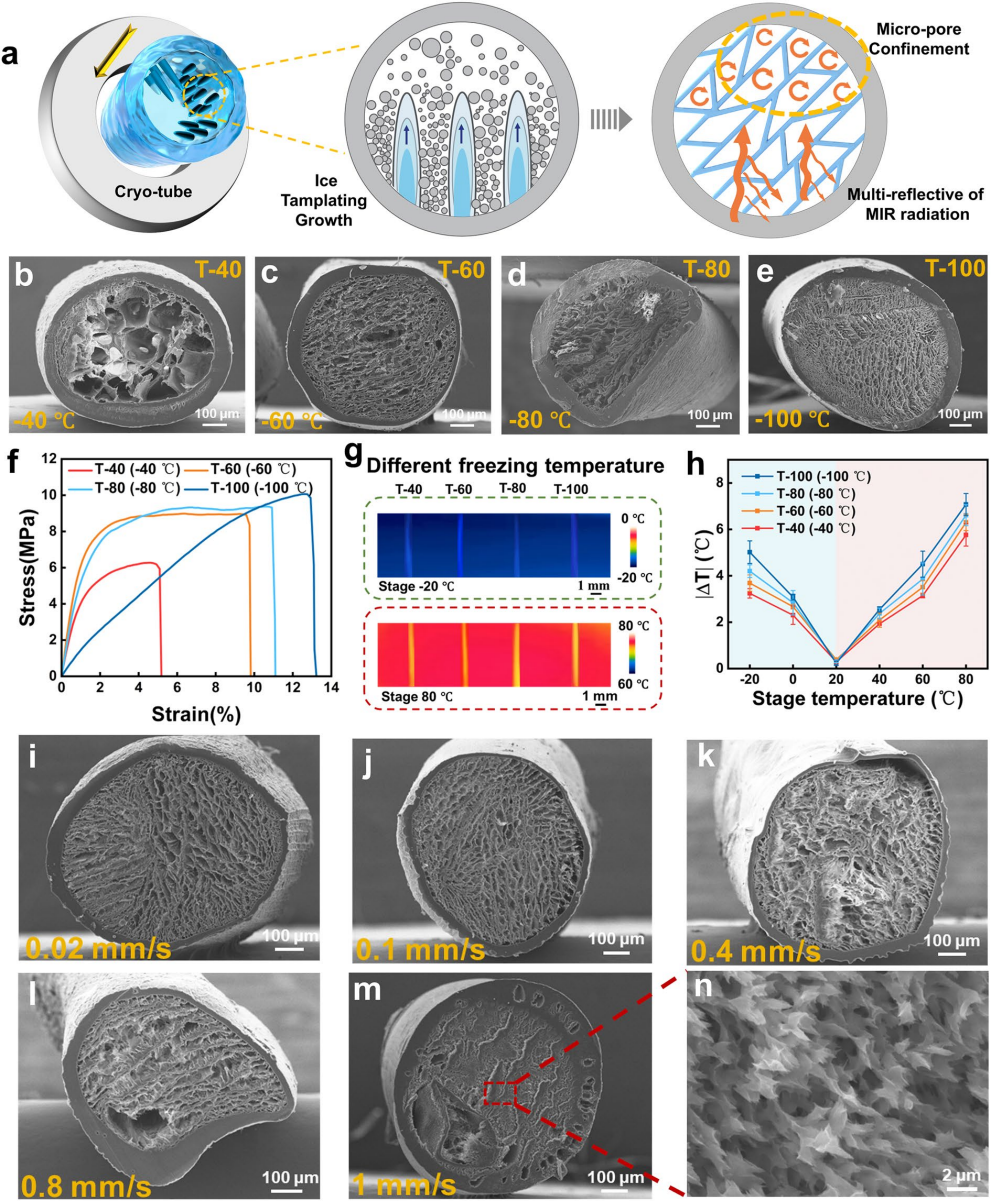
Effects of freezing temperature and rate on the microstructure and performance of SF@CL-AF. **a** Schematic illustration of the ice-crystal growth mechanism and the resulting lamellar structures, which enhance infrared a multi-reflective infrared effect and confine micropores. **b–e** Cross-sectional SEM images of aerogel fibers prepared at freezing temperatures of − 40, − 60, − 80, and − 100 °C, respectively. **f** Stress–strain curves of the aerogel fibers. **g** Infrared thermographic images of the four aerogel fibers at − 20 °C and 80 °C. **h** Absolute temperature difference (|ΔT|) between the aerogel fiber surface and the hot/cold stage. Cross-sectional SEM images of aerogel fibers fabricated under directional immersing rates of **i** 0.02 mm s^−1^, **j** 0.1 mm s^−1^, **k** 0.4 mm s^−1^, **l** 0.8 mm s^−1^, and **m** 1.0 mm s^−1^. **n** Magnified view of the boxed region in (**m**)

Freezing temperature mainly controls ice-crystal size and alignment, while immersing rate adjusts their growth, together shaping the hierarchical sheet-like porous structure aerogel. The structural characteristics are primarily regulated by the ice-templating process during directional freezing. Specifically, the cold-source temperature and precursor transit rate directly control the ice template morphology. The influence of freezing temperature and immersing rate on the structural and mechanical properties of the fibers were studied systematically (Fig. 3a). To specifically evaluate the influence of freezing temperature, aerogel fiber samples prepared via directional freezing with cold-source temperatures at − 40, − 60, − 80, and − 100 °C was designated as T-40, T-60, T-80, and T-100, respectively. The cross-sectional morphology, mechanical performance, and thermal insulation properties of the resulting fibers are presented in Fig. 3b–e. As the freezing temperature decreased, the internal pore size progressively decreased (S1), and the microstructure transitioned from a loosely irregular network to a densely packed, sheet-like architecture. At − 40 and − 60 °C, the pores were relatively large and irregularly distributed, resulting in a disordered and loosely organized structure. In contrast, at − 80 and − 100 °C, the pores exhibited a radially aligned, sheet-like morphology with moderate size and high uniformity, demonstrating excellent orientation and continuity. We suggest that this structural evolution involves thermodynamics and kinetics of ice-crystal formation during directional freezing. Lower freezing temperatures increase supercooling, which boosts crystal nucleation and limits growth, resulting in many small and uniform pores. Higher freezing temperatures favor ice-crystal growth over nucleation, leading to fewer but larger pores and a coarser structure. Similar temperature effects have been reported in other freeze-casting systems [42, 43].

This structural evolution directly correlates with the mechanical and thermal insulation behavior of the aerogel fibers. As the freezing temperature decreased from − 40 to − 100 °C, the tensile strength of the aerogel fibers increased from 7.30 to 10.53 MPa, while the elongation at break rose from 5.16% to 13.55% (Fig. 3f). This mechanical enhancement can be attributed to the rapid formation of fine ice crystals at low temperature, which promote a dense and more interconnected pore network. Such a microstructure contributes to improved structural integrity and more uniform stress distribution, thereby enhancing both strength and toughness.

Thermal insulation performance of the aerogel fibers was also evaluated by infrared thermography under heat (80 °C) and cold (− 20 °C) conditions (Fig. 3g). The absolute temperature difference (|ΔT|) between the fiber surface and the thermal stage increased with decreasing freezing temperature (Fig. 3h). Under the cold condition, |ΔT| increased from ~ 3.2 °C for T-40 to ~ 5.0 °C for T-100, while under the heat condition |ΔT| increased from ~ 5.8 °C for T-40 to ~ 7.0 °C for T-100. These results indicate that lower freezing temperatures improve thermal insulation performance of aerogel fibers due to the formation of a more uniform microporous structure that suppresses heat transfer.

In addition to freezing temperature, the immersing rate serves as a critical processing parameter that governs ice-crystal formation and porous structure development, thereby exerting a decisive influence on the formation of biomimetic sheet-like structures [44]. To systematically investigate the influence of immersing rate on the micro-nanostructure of the aerogel fiber core, directional freezing experiments were carried out by immersing the sample into the low-temperature freezing module at different rates (0.02, 0.1, 0.4, 0.8, and 1.0 mm s^−1^). At a slow immersing rate of 0.02–0.1 mm s^−1^, a well-aligned sheet-like porous structure formed in the fiber core (Fig. 3i, j). As increasing the immersing rate, the internal pore morphology became increasingly disordered (Fig. 3k, l). When the immersing rate exceeds 1.0 mm s^−1^, the core region transitioned into a disordered microporous structure. This transition is mainly attributed to random nucleation of ice crystals within low-temperature freezing module, which suppressed their directional growth (Fig. 3m). These results highlight the critical influence of freezing rate in guiding ice-crystal growth dynamics during the production of core–shell aerogel fibers. The obtained SF@CL-AF textile exhibits strong absorption in the 8–13 μm range, together with low transmittance and low reflectance (S3), indicating enhanced attenuation of infrared radiation within the hierarchical porous architecture. The sheet-like architecture formed at low immersing rates not only suppresses thermal conduction by introducing interfacial barriers but also significantly contributes to the regulation of radiative heat transfer through a multi-reflective effect.

### Geometry-Dependent Performance of Core–Shell Aerogel Fibers

**Fig. 4:**
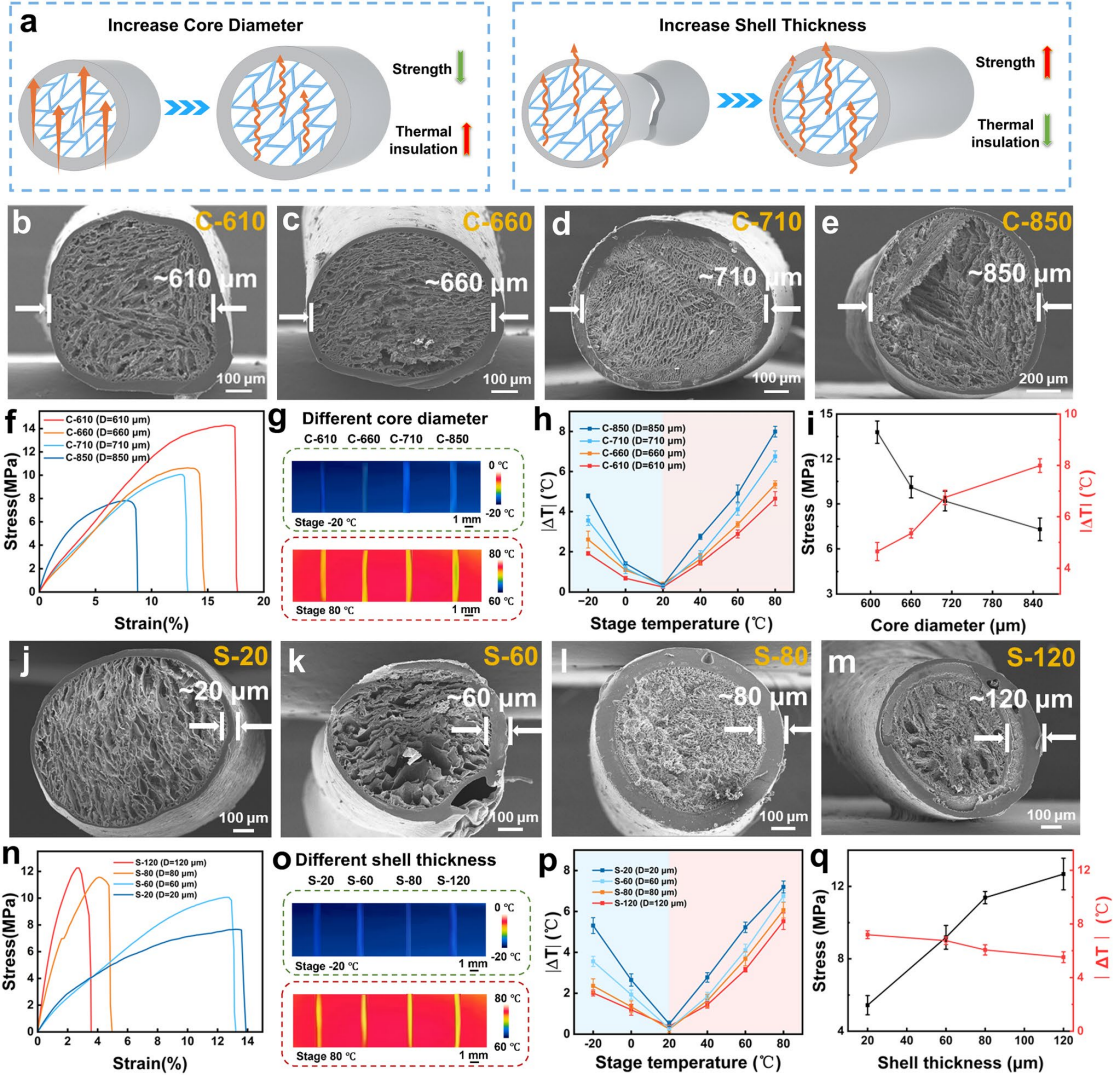
Influence of core diameter and shell thickness on the performance of aerogel fibers. **a** Schematic illustration of the effects of core on the thermal insulation, and shell thickness on both the mechanical strength and thermal insulation. Cross-sectional SEM images of aerogel fibers with various core diameters 611 ± 16 μm (**b**) 672 ± 40 μm (**c**) 749 ± 45 μm (**d**) and 880 ± 30 μm (**e**) and a uniform shell thickness of ~ 60 μm. **f** Stress–strain curves of aerogel fibers with various core diameters. **g** Infrared thermographic images of the four aerogel fibers at− 20 °C and 80 °C. **h** Scatter plot of the absolute temperature difference (|ΔT|) between the aerogel fiber surface and the hot/cold stage. **i** Fracture strength and thermal insulation performance as a function of core diameter. Cross-sectional SEM images of fibers with a fixed core diameter of ~ 710 μm and various shell thicknesses of 26 ± 7 μm (**j**), 58 ± 4 μm (**k**), 76 ± 4 μm (**l**), and 107 ± 11 μm (**m**). **n** Stress–strain curves of aerogel fibers with various shell thicknesses. **o** Infrared thermographic images of aerogel fibers at − 20 °C and 80 °C. **p** Scatter plot of the absolute temperature difference (|ΔT|) between the aerogel fiber surface and the hot/cold stage. **q** Fracture strength and thermal insulation performance as a function of shell thickness

Achieving an optimal balance between the mechanical robustness imparted by the dense cellulose shell and the thermal insulation provided by the aerogel core is key to enabling practical applications (Fig. 4a). To precisely control the core dimensions, coaxial nozzles with different inner diameters (0.20, 0.27, 0.30, and 0.60 mm) were used to prepare a set of aerogel fiber samples (denoted as C-610, C-660, C-710, and C-850) with tunable core diameters. The obtained aerogel core diameters were measured to be 611 ± 16, 672 ± 40, 749 ± 45, and 880 ± 30 μm, respectively. SEM images reveal that increasing the inner needle diameter led to a clear expansion of the core region, while the overall integrity of the core–shell structure was maintained (Fig. 4b–e). Furthermore, a well-aligned sheet-like porous core structure was maintained for all the samples, indicating that core size modulation does not disrupt the ice templating induced by directional freezing. As the core diameter increases from approximately 610 to 850 μm, the tensile strength decreases of the aerogel fibers decreased from 14.12 to 7.82 MPa, accompanied by a reduction in elongation at break from 17.42% to 8.59% (Fig. 4f). This decline in mechanical performance is likely attributed to the increased core-to-shell ratio, which weakens structural synergy and impairs load distribution across the fiber.

The thermal insulation performance of the aerogel fibers improved with increasing core diameter (Fig. 4g, h). Under cold conditions, |ΔT| increased form ~ 1.9 °C for C-610 to ~ 4.8 °C for C-850, while under heat conditions, |ΔT| increased from ~ 4.6 °C for C-610 to ~ 8.0 °C for C-850. In contrast, the average tensile strength decreased from ~ 13.8 to ~ 7.3 MPa with increasing core diameter from 610 to 850 μm (Fig. 4i). Considering the trade-off between thermal insulation and mechanical strength, a core diameter of ~ 710 μm was selected as the optimal parameter for the further testing. The shell thickness of core–shell aerogel fibers was investigated due to its influence on both the mechanical properties and thermal conduction behavior. A set of core–shell aerogel fibers were prepared with the shell thicknesses of 26 ± 7, 58 ± 4, 76 ± 4, and 107 ± 11 μm while maintain the core diameter the same. SEM images (Fig. 4j–m) show that as the shell thickness increases, the overall fiber diameter expands and the core–shell interface grows more distinct. Increasing shell thickness from approximately 20 to 120 μm leads to increased fiber tensile strength from 7.62 to 12.26 MPa, while the elongation at break decreased from 13.59% to 3.43% (Fig. 4n). These results indicate thicker shell could enhance structural integrity and mechanical robustness but reduce its flexibility.

Thermal insulation performance of aerogel fibers with different shell thicknesses was evaluated under cold (− 20 °C) and heat (80 °C) conditions (Fig. 4o). As shell thickness increased, the |ΔT| values gradually decreased (Fig. 4p). Under cold conditions, |ΔT| decreased from ~ 5.3 °C for S-20 to ~ 2.0 °C for S-120, while under heat conditions, |ΔT| decreased from ~ 7.2 °C for S-20 to ~ 5.5 °C for S-120. Meanwhile, the tensile strength increased from ~ 5.4 MPa for S-20 to ~ 12.7 MPa for S-120 (Fig. 4q). The reduced thermal insulation performance is mainly attributed to enhanced heat conduction through the increased cellulose shell. Considering the trade-off between thermal insulation and mechanical durability, a shell thickness of ~ 60 μm was selected for the following test.

### Continuous Production and Biodegradation of Aerogel Fibers

**Fig. 5:**
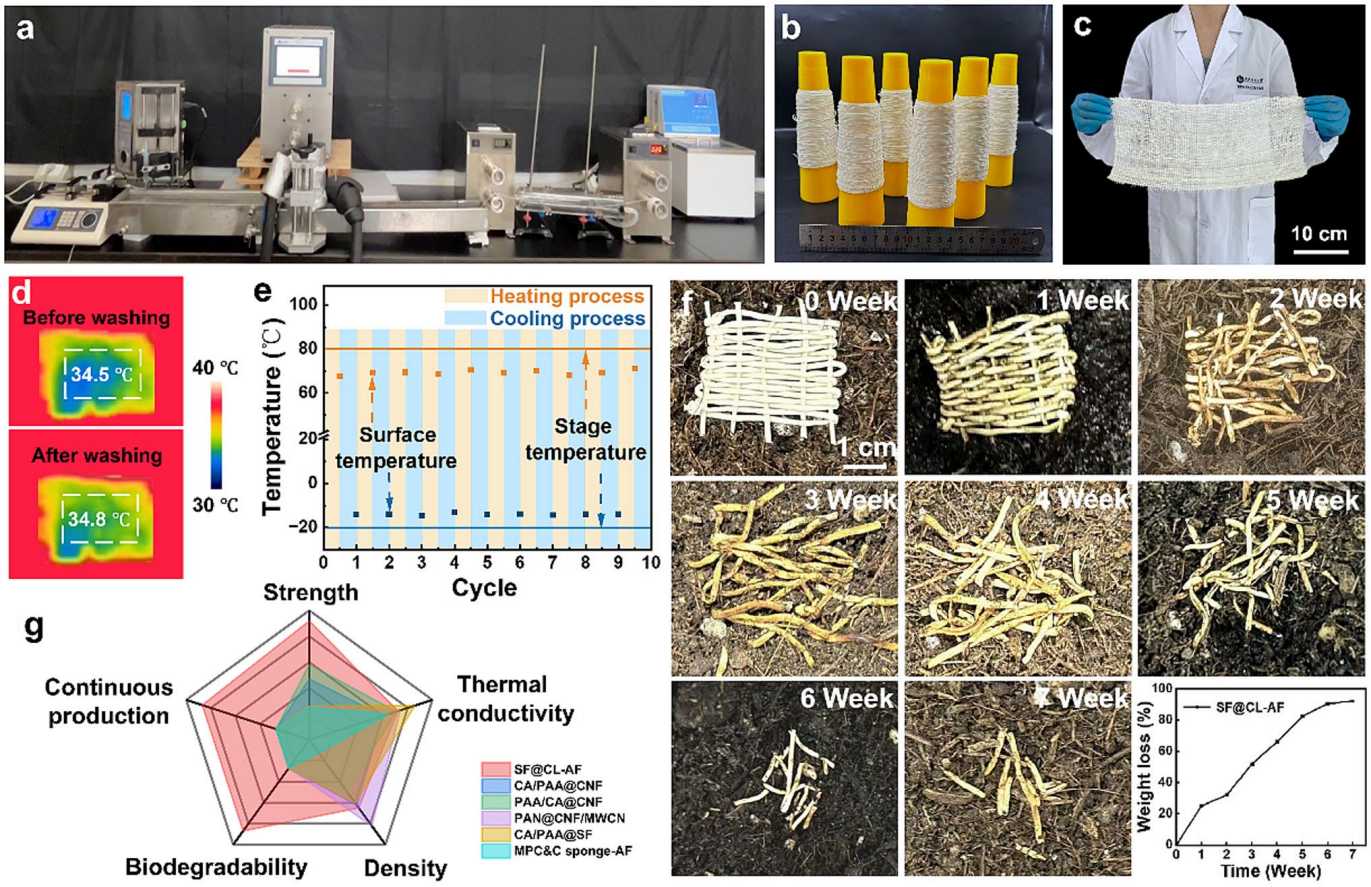
Continuous production and biodegradation of aerogel fibers. **a** Continuous production setup for SF@CL-AF. **b** Optical image of the as-prepared aerogel fiber spool. **c** Photograph of the fabricated aerogel fabric (40 × 25 cm^2^). **d** Infrared thermal images of the single-layer aerogel fabric at 40 °C heating plate before and after washing. **e** Surface temperature response of the aerogel fabric during repeated cycling from − 20 to 80 °C. **f** Biodegradability test of SF@CL-AF textile over 7 weeks. **g** Radar chart comparing five performance parameters of different coaxial aerogel fibers [6, 15, 45–47]

This work enabled the continuous, ambient-pressure production of all- biomass-based, high-performance core–shell aerogel fibers. Scalable production is achieved through the coordinated combination of directional ice templating, solvent-induced core gelation, and ambient drying, which collectively drive spontaneous shell encapsulation-as visualized in the panoramic setup image (Fig. 5a and Video S1). This process demonstrates scalability, as evidenced by the successful formation of multiple SF@CL-AF spools and large-area woven fabrics (Fig. 5b, c). The woven aerogel fabric exhibits stable thermal insulation after a washing cycle, with only a negligible surface temperature difference was observed, which was 34.5 °C before washing and 34.8 °C after washing on a 40 °C hot stage (Fig. 5d). Under dynamic thermal cycling from − 20 to 80 °C, the fabric surface temperature was − 14 and 70 °C, respectively (Fig. 5e), indicating stable thermal regulating performance. Biodegradability of the SF@CL-AF textiles was evaluated under simulated environmental conditions. The fiber underwent notable structural disintegration over time, achieving over 90% disintegration within 6 weeks (Fig. 5f). This result confirms its promising environmental compatibility. A radar chart compares the overall performance of various coaxial aerogel fibers in terms of tensile strength, thermal conductivity, density, biodegradability, and continuous production (Figs. 5g and S4, Tables S2 and S3) [6, 15, 45–47]. The SF@CL-AF offers a favorable balance of high mechanical strength, biodegradability, and scalability, while maintaining density and thermal conductivity comparable to previously reported counterparts.

## Conclusions

In this study, inspired by porous core–shell structure of polar bear hair, we developed a continuous and scalable strategy to fabricate all-biomass aerogel fibers with a self-encapsulated core–shell architecture, using silk fibroin as the core and cellulose as the shell. By integrating coaxial wet spinning, directional freezing, solvent exchange, and ambient-pressure drying, both the core diameter and shell thicknesses are readily tunable and the sheet-like porous structure is precisely controllable. This enables an optimal balance between lightweight thermal insulation and mechanical robustness of the aerogel fiber. The optimized aerogel fiber achieves a high porosity of 79.78%, a low density of 0.193 ± 0.01 g cm⁻^3^, and a low thermal conductivity of 52.4 ± 4.2 mW m⁻^1^ K⁻^1^. Notably, the aerogel fiber-based fabrics maintain effective thermal insulation under cold-water immersion and after washing, while also exhibiting flexibility, dyeability, and biodegradability. Beyond wearable thermal management, this work provides a scalable and eco-friendly pathway toward sustainable aerogel textiles for extreme-environment protection. Nevertheless, further studies on long-term durability, repeated washing stability, and practical large-scale textile integration are still required for real-world applications.

## Supplementary Information

Below is the link to the electronic supplementary material.

Supplementary file1 (DOCX 1002 KB)

Supplementary file2 (MP4 1855 KB)
