# Supplementary material for: Continuous Fabrication of Bioinspired All-Biomass Aerogel Fibers for Thermal Insulation
Source: Nanomicro Lett. 2026 Aug 10;19:23. doi: 10.1007/s40820-026-02332-3 (PMC13454090; doi:10.1007/s40820-026-02332-3)
Supplement: Supplementary file 1 [file 40820_2026_2332_MOESM1_ESM.docx]

Supporting Information for

**Continuous Fabrication of Bioinspired All-Biomass Aerogel Fibers for Thermal Insulation**

Meiling Zhou^1^, Qiaoling Xue^1^, Lulu Fu^1^, Beini Zeng^1^, Shengnan Ouyang^1^, Shouwei Zhang^1^, Jinming Zhang^2,*^, Qingtao Liu^1,3^, Xungai Wang^3^ and Jinfeng Wang^1,*^

^1^ National Local Joint Laboratory for Advanced Textile Processing and Clean Production, Wuhan Textile University, Wuhan 430200, P. R. China

^2^ CAS Key Laboratory of Engineering Plastics, Institute of Chemistry, Chinese Academy of Sciences (CAS), Beijing 100190, P. R. China

^3^ College of Textile Science and Engineering (International Institute of Silk), Zhejiang Sci-Tech University, Hangzhou 310023, P. R*.* China

* Corresponding authors. E-mail: Jinfeng.wang@wtu.edu.cn (Jinfeng Wang); zhjm@iccas.ac.cn (Jinming Zhang)

**S1 Supplementary Text**

***S1 Thermal conductivity calculation***

The thermal conductivity of textile samples was measured using a steady-state device, following the method reported by Wu et al. [S1] The thermal conductivity of textile samples was measured using a steady-state device. The sample was sandwiched between two aluminum blocks, with the bottom block heated by a hot stage as the heat source. At thermal equilibrium, the steady-state temperature difference (T_1_, T_2_) between the two blocks was recorded using an IR camera. The effective heat transfer rate was calibrated by removing the sample and monitoring the free cooling process of the top block, allowing the heat dissipation rate to be determined from its cooling slope. Based on the heat balance and geometric factors of the Al blocks, the textile’s thermal conductivity (λ) was calculated according to the following relation:

$$\lambda=\frac{mc_{p}\delta(ab+2ac+2bc)}{2ab(ab+ac+bc)(T_{1}-T_{2})}\frac{dT}{dt}$$

where m, c_p_, and δ are the mass, specific heat capacity, and thickness of the sample, and a, b, c are the dimensions of the Al block.

**S2 Supplementary Figures**

**Fig. S1** **a** Mercury intrusion pore-size distribution curves of samples prepared at different freezing temperatures. **b** Average pore diameters calculated from MIP analysis.

**Fig. S2** **a** Magnified view of core in cellulose-core/SF-shell fiber. **b** Magnified view of core in SF-core/cellulose-shell fiber fabricated without directional freezing. **c** Magnified view of core in SF-core/cellulose-shell fiber fabricated without ethanol-curing.

**Fig. S3** **a** Infrared transmittance of SF and Cel films, respectively. The film thicknesses were selected to be consistent with the aerogel fabric. **b** Absorption, transmittance, and reflectance spectra of the SF@CL aerogel fabric in the 2.5–20 μm range. **c** Thermal insulation performance of SF@CL aerogel fabric with or without radiation heat.

The aerogel fabric exhibits high infrared absorptance with low transmittance and low reflectance across the measured wavelength range. The porous lamellar structure promotes multiple internal reflections and prolongs the infrared propagation pathway, thereby promoting infrared attenuation and suppressing radiative heat transfer. Moreover, the slight temperature difference between the two conditions suggests an intrinsic radiative regulation capability, enabling effective suppression of infrared radiative heat transfer and contributing to the textile’s excellent overall thermal insulation performance.

**Fig. S4** Comparison of thermal conductivity and stress for different coaxial aerogel fibers.

**S3 Supplementary Tables**

**Table S1** Thickness and thermal conductivity of various textile materials.

| Material | Thickness (mm) | T_surface_ (℃) | Λ (mW/m·K) |
| --- | --- | --- | --- |
| PET | 2.3±0.09 | 38.4±0.2 | 100.1±12.2 |
| Nylon | 2.27±0.02 | 37.7±0.1 | 82.5±3.1 |
| Cotton | 2.40±0.03 | 37.2±0.1 | 71.9±7.1 |
| Wool | 2.36±0.04 | 36.4±0.3 | 63.0±7.4 |
| SF@CL-AF | 2.21±0.04 | 32.8±0.4 | 52.4±4.2 |

**Table S2** Summary of mechanical strength, thermal conductivity, density, biodegradability, and continuous production capability of different coaxial aerogel fibers

| Score Range | Biodegradability | Continuous Production Capability |
| --- | --- | --- |
| 0-2 | Non-degradable synthetic materials; no biodegradation reported. | Laboratory-scale, discontinuous or multi-step processes; not suitable for continuous production. |
| 3-5 | Very slow or partial degradation over long periods (>3 months) under natural conditions. | Semi-continuous or partially automated process; limited scalability. |
| 6-8 | Noticeable biodegradation within weeks to months; partial weight loss or surface erosion observed. | Continuous production demonstrated on lab scale; process stability moderate. |
| 9-10 | Rapid biodegradation within days to weeks; full disintegration or high mass loss. | Fully continuous, stable, and scalable process demonstrated |

The biodegradability of the SF@CL-AF was experimentally evaluated in this work, and its continuous production capability was determined from the demonstrated scalable spinning process. In contrast, the other fibers reported in the literature were neither continuously produced nor experimentally tested for biodegradability, as they were generally fabricated via batch or multi-step methods. For comparability, biodegradability and continuous production capabilities were semi-quantitatively evaluated on a 0-10 scale, where higher scores indicate faster environmental degradation and greater feasibility of scalable continuous fabrication. Materials without available data were assigned a default score of 2 (“None”). The detailed scoring criteria and performance data are presented below.

**Table S3** Summary of mechanical strength, thermal conductivity, density, biodegradability, and continuous production capability of different coaxial aerogel fibers

| Material | SF@CL-AF | CA/PAA@CNF [S2] | PAA/CA@CNF  [S3] | PAN@CNF/  MWCNT  [S4] | CA/PAA@SF [S5] | MPC&C sponge-AF [S6] | |
| --- | --- | --- | --- | --- | --- | --- | --- |
| Strength (MPa) | 9.19 | 4.7 | 5.83 | 2.62 | 2.6 | 2.5 |  |
| Thermal conductivity  (W·m⁻¹·K⁻¹) | 0.052 | 0.07 | 0.054 | 0.049 | 0.031 | 0.064 |  |
| Density (g·cm^-3^) | 0.19 | 0.2 | 0.25 | 0.121 | 0.21 | None |  |
| Biodegradability  Week / Score | 7 / 8 | None / 2 | None / 2 | None / 2 | None / 2 | None / 2 |  |
| Continuous production / Score | 1 mm·s^-1^ / 8 | None / 2 | None / 2 | None / 2 | None / 2 | None / 2 |  |

**S4 Supporting Videos**

**Video S1** Illustrates the continuous, ambient-pressure fabrication of the biomimetic core-shell aerogel fiber.

**Supplementary References**

[S1] M. Wu, Z. Shao, N. Zhao, R. Zhang, G. Yuan et al., Biomimetic, knittable aerogel fiber for thermal insulation textile. Science **382**(6677), 1379–1383 (2023). <https://doi.org/10.1126/science.adj8013>
[S2] J. Zhou, Y.-L. Hsieh, Nanocellulose aerogel-based porous coaxial fibers for thermal insulation. Nano Energy **68**, 104305 (2020). <https://doi.org/10.1016/j.nanoen.2019.104305>
[S3] H. Sun, W. Mu, X. Cui, Z. Xu, T. Zhang et al., Polymer-encapsulated aerogel fibers prepared *via* coaxial wet spinning with stepwise coagulation for thermal insulation. ACS Appl. Polym. Mater. **5**(1), 552–559 (2023). <https://doi.org/10.1021/acsapm.2c01648>
[S4] W. Mu, S. University, H. Cao, S. University, X. Cui et al., Multifunctional polymer-encapsulated aerogel fibers with thermal insulation, active heating, and phase change energy storage abilities. ACS Appl. Polym. Mater. **6**(24), 15162–15171 (2024). <https://doi.org/10.1021/acsapm.4c02756>
[S5] H. Yang, Z. Wang, Z. Liu, H. Cheng, C. Li, Continuous, strong, porous silk fibroin-based aerogel fibers toward textile thermal insulation. Polymers **11**(11), 1899 (2019). <https://doi.org/10.3390/polym11111899>
[S6] J. Huang, H. Wu, Z. Su, Cellulose hierarchical sponge-aerogel fibers*via*ionic liquid-assisted coaxial wet spinning: lightweight architectures for gas detection and adaptive thermal management. J. Mater. Chem. A **13**(29), 23882–23892 (2025). <https://doi.org/10.1039/d5ta01915j>
